# Transcutaneous electric nerve stimulation over acupoints for chronic obstructive pulmonary disease: Protocol for a systematic review and meta-analysis

**DOI:** 10.1097/MD.0000000000011199

**Published:** 2018-06-22

**Authors:** Jia-Jia Wang, Yang Xie, Hu-Lei Zhao, Wei-Hong Han, Xiao-Chun Wang

**Affiliations:** a Collaborative Innovation Center for Respiratory Disease Diagnosis and Treatment and Chinese Medicine Development of Henan Province, Henan University of Chinese Medicine, Zhengzhou; b Dongzhimen Hospital, Beijing University of Chinese Medicine, Beijing; c Department of Respiratory Diseases, The First Affiliated Hospital of Henan University of Chinese Medicine, Zhengzhou, China.

**Keywords:** chronic obstructive pulmonary disease, meta-analysis, protocol, systematic review, transcutaneous electric nerve stimulation over acupoints

## Abstract

**Background:**

There is a limited evidence concerning the efficacy of transcutaneous electric nerve stimulation over acupoints (Acu-TENS) for chronic obstructive pulmonary disease (COPD). Thus, this review aims to systematically determine the effect of Acu-TENS on COPD.

**Methods:**

PubMed, Embase, The Cochrane Library, Web of Science, Chinese Biomedical Literature Database, China National Knowledge Infrastructure, Chongqing VIP, and Wanfang Data will be searched from their inception to May 10, 2018. Randomized controlled trials that evaluated the effect of Acu-TENS on patients with COPD will be included. The primary outcome measures will include 6-minute walk distance and dyspnea visual analog scale scores. The secondary outcome measures will include lung function and St George's Respiratory Questionnaire. Study selection, data extraction, and risk of bias assessment will be independently undertaken, respectively. Statistical analysis will be conducted by RevMan software (version 5.3).

**Results:**

This systematic review will provide a detailed summary of current evidences related to the efficacy of Acu-TENS in improving exercise capacity, breathlessness, quality of life, and lung function of patients with COPD.

**Conclusion:**

This evidence may be useful to clinicians, patients, and health policy makers with regard to the use of Acu-TENS in the treatment of COPD.

**Ethics and dissemination:**

This review will not gather original data; hence, ethical approval is not required. The results will be disseminated through a peer-reviewed publication or conference presentations.

## 1 Introduction

Chronic obstructive pulmonary disease (COPD), characterized by persistent respiratory symptoms and airflow limitation, is a common, preventable, and treatable disease, and is a leading cause of morbidity and mortality worldwide with a substantial and increasing social and economic burden.^[1–3]^ It is projected to be the third leading cause of death worldwide by 2030, and will remain a significant public health problem for the foreseeable future.^[4,5]^ Dyspnea, exercise limitation, lung function, and health status impairment broadly exist in patients with COPD, and therefore effective measures should be taken to relieve symptoms, improve exercise capacity, delay the decline of lung function, and improve quality of life based on an individualized assessment of disease.^[1]^ At present, although appropriate pharmacologic therapies have been proven to be effective in relieving symptoms, reducing the frequency and severity of exacerbations, and improving health status and exercise capacity of patients with COPD; however, many gaps are still to be filled.^[1,5]^ For example, the cost and adverse effects of pharmacologic therapies can never be ignored.

Transcutaneous electric nerve stimulation (TENS), a western treatment modality, is the use of electric current produced by a device to stimulate the nerves for therapeutic purposes. TENS over acupoints (Acu-TENS) is an integrative intervention merging TENS with acupuncture, a traditional Chinese treatment.^[6]^ As for traditional acupuncture, it is necessary to insert a needle into acupoints for stimulation. By comparison, Acu-TENS is a noninvasive intervention, which induces acupoint therapeutic effects through application of surface electrodes over the acupoints. In recent years, it has been widely used to treat COPD. However, there is a limited evidence concerning its efficacy for COPD. Thus, this review aims to systematically determine the efficacy of Acu-TENS for COPD, providing a scientific evidence for clinical decision.

## 2 Methods and analysis

### 2.1 Criteria for considering studies

Randomized controlled trials evaluating the effect of Acu-TENS on COPD will be included.

We will include participants with a clinical diagnosis of COPD according to the global initiative for chronic obstructive lung disease, but we will exclude those who had an acute exacerbation within 4 weeks before the study.

We will include trials where Acu-TENS was compared to placebo/sham Acu-TENS.

Primary outcome measures will include 6-minute walk distance^[7]^ and dyspnea visual analog scale scores.^[8,9]^ Secondary outcome measures will include lung function (forced expiratory volume in 1 second and forced vital capacity included) and St George's Respiratory Questionnaire.^[10]^

### 2.2 Literature search

PubMed, Embase, The Cochrane Library, Web of Science, Chinese Biomedical Literature Database, China National Knowledge Infrastructure, Chongqing VIP, and Wanfang Data will be searched from their inception dates to May 10, 2018. Reference lists of eligible studies and previous systematic reviews will also be reviewed to identify further eligible studies. We have developed detailed search strategies for each electronic database without language restrictions to identify all eligible studies. The search strategy for PubMed is shown in Table 1.

**Table 1:**
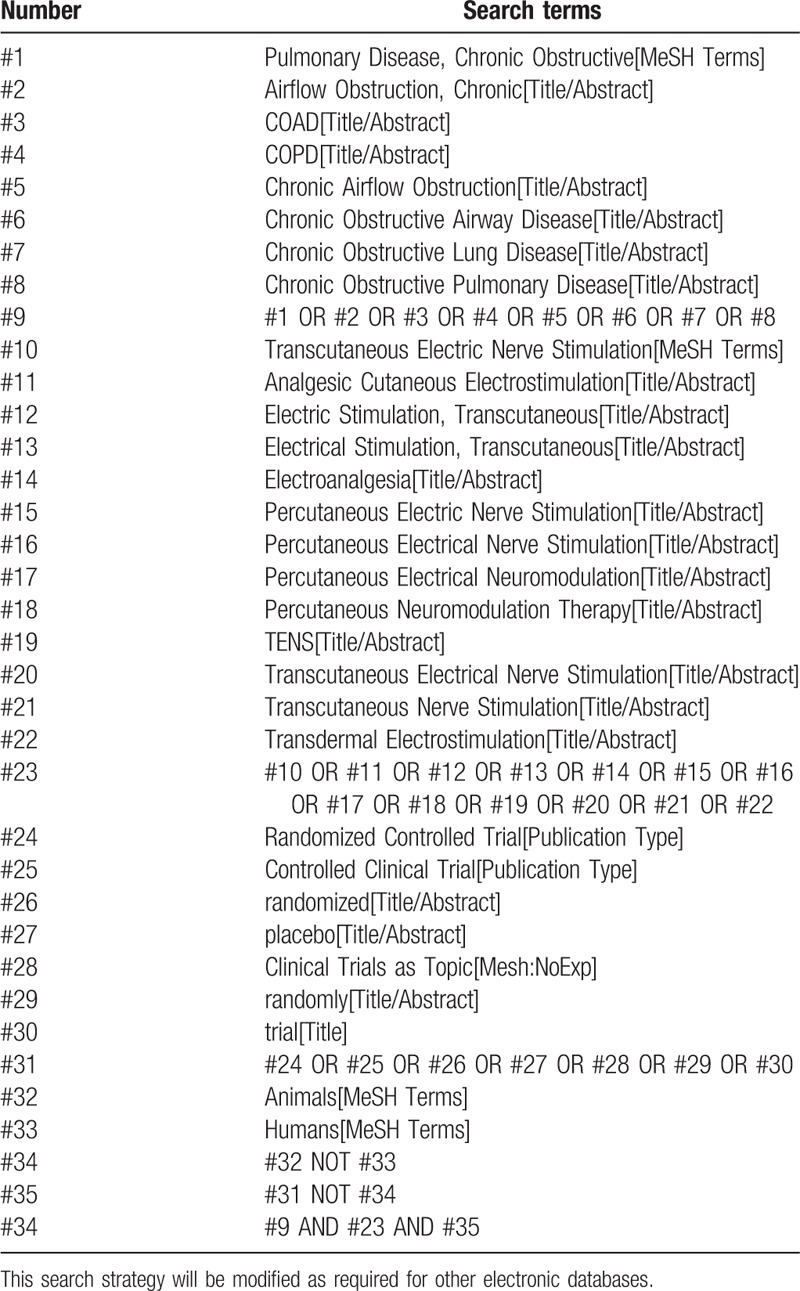
Search strategy for PubMed.

### 2.3 Study selection

**Figure 1:**
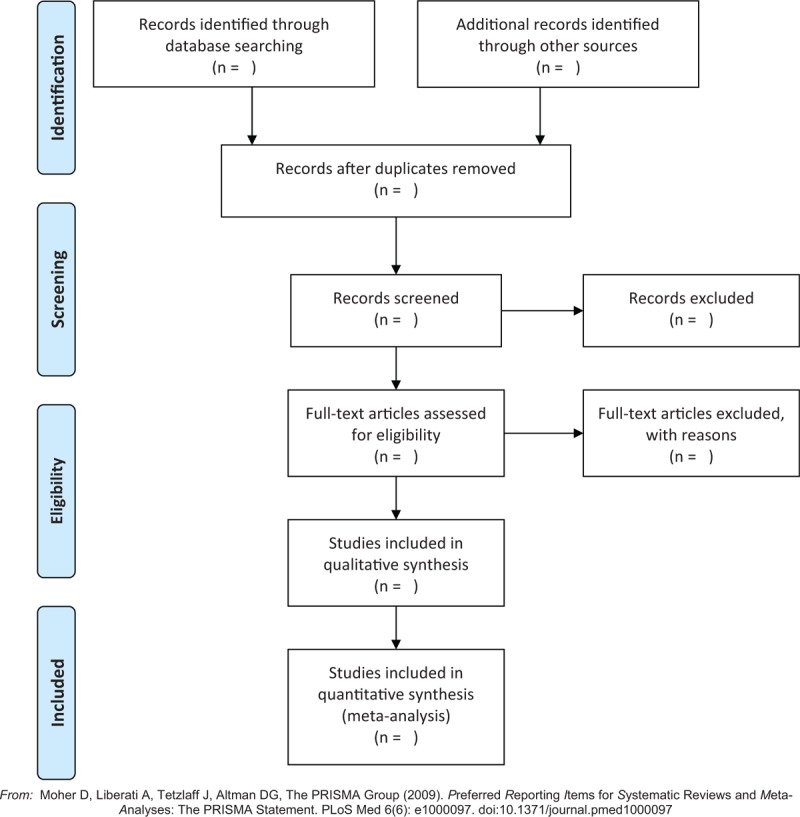
Study flow diagram.

Two review authors (J-JW and H-LZ) will independently examine titles and abstracts retrieved from the search, and select all potentially eligible studies. Then these full-text copies will be obtained and the same review authors will further review them independently against the inclusion and exclusion criteria. Any disagreements will be resolved through discussion or, if required, through consultation with a third review author (YX). The study flow diagram is described in Figure 1.^[11]^

### 2.4 Data extraction

Data extraction will be independently conducted by 2 review authors (J-JW and H-LZ) using a standardized data extraction sheet, and the following information will be collected: authors, year of publication, region, study design, participants, intervention (acupoints included), comparator and outcomes of included studies. A third review author (YX) will act as an arbiter if differences in opinion occur between J-JW and H-LZ.

### 2.5 Risk of bias assessment

Methodologic quality will be evaluated using the following criteria as described in the Cochrane Handbook of Systematic Reviews of Interventions^[12]^:

- Sequence generation
- Allocation sequence concealment
- Blinding of participants and personnel
- Incomplete outcome data
- Selective outcome reporting
- Other potential sources of bias

Two review authors (J-JW and H-LZ) will independently assess each study, with a third review author (YX) acting as an arbiter if disagreements arise.

### 2.6 Statistical analysis

Statistical analysis will be conducted by RevMan software (version 5.3).^[13]^ We will express the effects using odds ratio with 95% confidence intervals (CIs) for dichotomous outcomes and mean difference with 95% CI for continuous outcomes. If the data cannot be combined into a meta-analysis, we will summarize it in the text. When standard deviation (SD) of the change of the outcome measures are absent, we will substitute for them the mean SD of other included studies. We will exclude the studies from the analysis in which only medians and percentiles are available, and there are no means of calculating the mean change. χ^2^ test will be used to estimate heterogeneity, and further analysis will be performed using *I*^2^ test. We will regard heterogeneity as substantial when *I*^2^ is >50% or a low *P*-value (<.10) is reported for the χ^2^ test.^[14]^ A random-effect model will be used if heterogeneity is substantial, whereas a fixed-effect model will be used if heterogeneity is found to be not statistically significant. When 10 or more studies are included in the meta-analysis, we will investigate potential publication bias using a funnel plot. If necessary, we will conduct subgroup analysis to assess whether the treatment effects are different in different subgroups.

### 2.7 Grading the quality of evidence

The Grading of Recommendations Assessment, Development and Evaluation approach will be used to describe the overall quality of the outcomes.^[15]^ The quality of outcome measures will be categorized as high, moderate, low, and very low.

## 3 Discussion

To our knowledge, this is the first systematic review to investigate the efficacy of Acu-TENS for COPD. It will provide a detailed summary of the current evidences related to the efficacy of Acu-TENS in improving exercise capacity, breathlessness, quality of life, and lung function of patients with COPD. This evidence may be useful to clinicians, patients, and health policy makers with regard to the use of Acu-TENS in the treatment of COPD.

## Author contributions

J-JW drafted the protocol. YX conceived this study, developed the search strategy and revised the manuscript. J-JW and H-LZ will independently screen the potential studies, extract data from the included studies, and assess the risk of bias. W-HH and X-CW will conduct statistical analysis. All authors have read and approved the final manuscript for publication.

**Conceptualization:** Yang Xie.

**Data curation:** Jiajia Wang, Hulei Zhao.

**Formal analysis:** Weihong Han, Xiaochun Wang.

**Writing – original draft:** Jiajia Wang.

**Writing – review & editing:** Yang Xie.
